# Complete Genome Sequences of Five SO-1-Like *Siphoviridae* Bacteriophages That Infect *Enterobacteriales*

**DOI:** 10.1128/mra.01224-21

**Published:** 2022-03-16

**Authors:** Madelyn G. Purnell, Kyle Andersen, Adam Bell, Jared T. Briscoe, Hannah M. F. Brown, Emilee L. Carr, Justen Doney, Parker F. Folsom, Cheyanne Green, Evan H. Harris, Elisa Huhem, R. Matthew Jensen, Liberty Johnson, Carter Jones, Andrew S. Lambert, Emily Loertscher, Colleen R. Newey, Matthew Porter, Jonah Rallison, Ruchira Sharma, Carson Sork, Silvia Soule, Jared B. Stewart, Tyson Stoker, Sadie Tayler, Daniel W. Thompson, Trever L. Thurgood, Jamison Walker, Donald P. Breakwell, Sherwood R. Casjens, Julianne H. Grose

**Affiliations:** a Department of Microbiology and Molecular Biology, Brigham Young University, Provo, Utah, USA; b Department of Biology, University of Utah, Salt Lake City, Utah, USA; c Division of Microbiology and Immunology, Department of Pathology, University of Utah School of Medicine, University of Utah, Salt Lake City, Utah, USA; Queens College CUNY

## Abstract

The *Enterobacteriales* order is composed of Gram-negative bacteria that range from harmless symbionts to well-studied pathogens. We announce complete genome sequences of five related SO-1-like *Enterobacteriales* bacteriophages (also known as the *Dhillonvirus* genus) isolated from wastewater that infect Escherichia coli (Opt-212, Over9000, Pubbukkers, and Teewinot) or Shigella boydii (StarDew).

## ANNOUNCEMENT

The *Enterobacteriaceae* family of bacteria includes well-known model organism species and clinical pathogens such as the closely related species Escherichia coli and Shigella boydii. These bacteria are known to cause hospital-acquired infections and to acquire antibiotic resistance, making phage therapy a possible alternative treatment. Here, the isolation, complete genome sequences, and annotation of five SO-1-like siphophages that infect E. coli and S. boydii are presented.

All five bacteriophages were isolated from wastewater treatment plants in the western United States by propagation in enrichment culture on E. coli BW25113 (1) or S. boydii Ewing NCTC 12985 (ATCC 8700) in LB medium at 37°C. Enrichment cultures were centrifuged to pellet bacteria, and the supernatant was incubated with bacteria overnight and plated in LB top agar. Bacteriophages were then isolated through a minimum of three successive single-plaque purifications by picking a plaque, incubating it with a fresh bacterial overnight culture, and replating it in LB top agar. Lysates (>10^8^ PFU/mL) were prepared by incubating a plaque with diluted bacterial overnight culture in LB medium for 48 h. Phage genomic DNA was purified from the high-titer lysates using a phage DNA isolation kit (Norgen Biotek, Canada). Genomic DNA was prepared for paired-end sequencing with unique barcodes using either the Illumina TruSeq Nano DNA library preparation kit (Opt-212 and StarDew, which underwent 250-bp paired-end Illumina HiSeq 2500 sequencing) or the NEBNext Ultra II DNA library preparation kit (the other three phages, which underwent 150-bp paired-end sequencing with the Illumina iSeq100 system). All contigs were trimmed and assembled using the preset *de novo* assembly function of Geneious v.R11 for HiSeq data and v.8.0.5 for iSeq data (2) and subsequently were annotated using DNA Master (3) and GeneMarkS (4). All software was used with default settings. All five bacteriophages circularized during *de novo* assembly and share >90% nucleotide sequence identity with bacteriophage SO-1 (GenBank accession number GQ502199) by BLASTN (5). The phage sequencing data assembled into circles, suggesting terminally redundant virion chromosomes, and analysis of raw reads from phage Opt-212 using PhageTerm predicted headful packaging (6); therefore, all phages were oriented with the small terminase as the first gene.

Bacteriophage SO-1, which infects Sodalis glossinidius, was chosen as the protype for a virulent phage cluster (7) and has also been assigned to the *Dhillonvirus* genus defined by the International Committee on Taxonomy of Viruses (ICTV). GenBank currently contains genome sequences of over 45 bacteriophages that belong to this cluster and are reported to infect *Sodalis*, Escherichia, *Shigella*, or *Edwardsiella*; however, this virus group is generally understudied, with relatively few reports of phage characterization (8, 9). Further study of these phages will advance the understanding of this diverse and abundant phage cluster, whose members are capable of infecting a range of bacterial hosts.

### Data availability.

The accession numbers for all five bacteriophages are found in Table 1.

**TABLE 1:** Sequencing summary and basic properties of five SO-1-like *Siphoviridae* bacteriophages belonging to the *Dhillonvirus* genus

Phage name	GenBank accession no.	SRA accession no.	Total no. of reads	Sequencing coverage (range [mean]) (×)	Genome length (bp)	GC content (%)	Sewage sampling location coordinates
vB_EcoS_Opt-212	OL770278	SRR17231354	18,605	781–3,240 (1,298.5)	44488	54.4	37.6879N, 112.4702W
vB_EcoS_Over9000	OK499985	SRR17231364	191,821	264–1,020 (650.1)	44583	54.6	40.3462N, 111.7786W
vB_EcoS_Pubbukkers	OK499988	SRR17231389	244,496	571–1,351 (829.1)	44476	54.6	40.1643N, 111.4414W
vB_EcoS_Teewinot	OK499993	SRR17231365	145,536	154–845 (526.1)	41800	54.5	40.0608N, 111.7320W
vB_SboS_StarDew	OL615010	SRR17231375	23,057	20–87 (49.1)	44715	54.7	37.3382N, 121.8863W
